# Dual Mechanism for the Emergence of Synchronization in Inhibitory Neural Networks

**DOI:** 10.1038/s41598-018-29822-8

**Published:** 2018-07-30

**Authors:** Ashok S. Chauhan, Joseph D. Taylor, Alain Nogaret

**Affiliations:** Department of Physics, University of Bath, Bath, BA2 7AY UK

## Abstract

During cognitive tasks cortical microcircuits synchronize to bind stimuli into unified perception. The emergence of coherent rhythmic activity is thought to be inhibition-driven and stimulation-dependent. However, the exact mechanisms of synchronization remain unknown. Recent optogenetic experiments have identified two neuron sub-types as the likely inhibitory vectors of synchronization. Here, we show that local networks mimicking the soma-targeting properties observed in fast-spiking interneurons and the dendrite-projecting properties observed in somatostatin interneurons synchronize through different mechanisms which may provide adaptive advantages by combining flexibility and robustness. We probed the synchronization phase diagrams of small all-to-all inhibitory networks *in-silico* as a function of inhibition delay, neurotransmitter kinetics, timings and intensity of stimulation. Inhibition delay is found to induce coherent oscillations over a broader range of experimental conditions than high-frequency entrainment. Inhibition delay boosts network capacity (ln2)^−*N*^-fold by stabilizing locally coherent oscillations. This work may inform novel therapeutic strategies for moderating pathological cortical oscillations.

## Introduction

The synchronization of electrical activity in the brain has been studied for several years to understand the mechanisms underpining cognition^1,2^ and memory consolidation^3^. The *γ*-oscillations of cortical micro-circuits are thought to be initiated by networks of parvalbumin^4,5^ or somatostatin interneurons^6^ which entrain principal cells^7–9^. These two neuron sub-classes differ in their physiological characteristics and may have adapted to exploit specific nonlinear properties. An understanding of these properties and their functional advantages is now needed. Computational models have been used to test neuronal synchronization through the interneuron gamma (ING) mechanism^10,11^, the pyramidal interneuron gamma (PING) mechanism^8,12,13^, the action of both excitatory and inhibitory synapses^14–17^ and the modulation of long range inhibition by local dendritic gap junctions^18–23^, which have been derived from tonic current stimulation. Mutually inhibitory networks, however, are chaotic systems which encode the timings of current stimuli in cyclical paths of sequentially discharging neurons^24,25^. These networks are therefore expected to exhibit abrupt transitions between modes of oscillation when both the timings and amplitudes of stimuli are varied^26–29^. This is reminiscent of phase transitions in systems with many degrees of freedom whose sensitivity to interactions makes them difficult to predict from first principles. Recent advances in neuromorphic engineering^30,31^ allow such phase transitions to be measured in physical networks and are the only way to integrate complex multivariate stimuli in real time^32,33^ without compromise on model accuracy, size or complexity. A further merit of using neuromorphic systems is to demonstrate the robustness of the large number of stable modes of oscillation which we observe against noise and network imperfections. In particular, the maximum network capacity is found to be robust against synaptic noise, component-to-component fluctuations and other experimental deviations of relevance to cortical networks. In this way, we establish inhibition delay and high frequency entrainment as dual mechanisms providing robust and tuneable synchronization.

## Results

**Figure 1:**
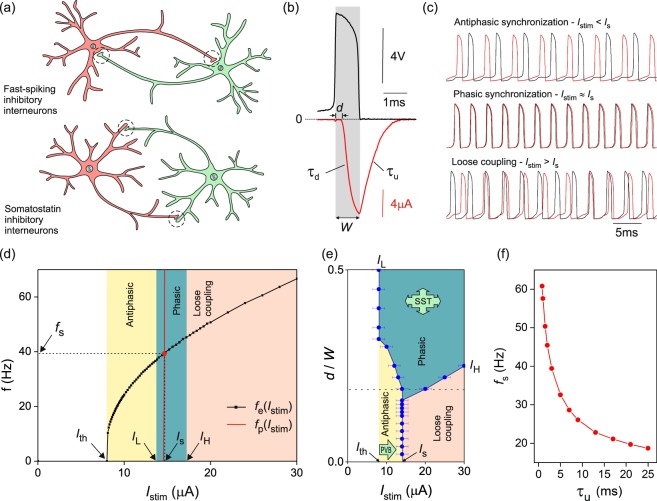
Synchronization of a pair of mutually inhibitory neurons and its dependence on synaptic kinetics. (**a**) Fast-spiking soma-projecting and somatostatin dendrite-projecting interneurons. Synapses located on dendrites effectively delay the inhibition of the postsynaptic neuron by 0– 800 *μ*s. (**b**) Inhibitory postsynaptic current (red line) evoked by a presynaptic action potential (black line) applied to a VLSI synapse. Synaptic kinetics: inhibition delay *d*, neurotransmitter docking time *τ*_*d*_, undocking time *τ*_*u*_, and spike width *W*. (**c**) Membrane voltage oscillations of mutually inhibitory neurons below, at, and above the synchronization current, *I*_*s*_. *τ*_*u*_ = 1.5 ms. (**d**) Frequency-current dependence of a VLSI neuron (square symbols) and frequency-current dependence of phase-locked oscillations (red line). Their intercept gives the frequency (*f*_*s*_) and current (*I*_*s*_) of phasic oscillations. Domains of synchronized oscillations at *d* = 0.2*W* (vertical bands). (**e**) Phase diagram of synchronization in the *d* − *I*_*stim*_ plane where delay *d* is normalised by the spike width *W*. Two alternative mechanisms contribute to synchronization in local inhibitory networks: a change in current stimulation (PVB: parvalbumin neuron-type synchronization) and an increase in inhibition delay (SST: somatostatin neuron-type synchronization). (**f**) Frequency of phasic oscillations as a function of the decay time of the postsynaptic current.

We built analog silicon models of all-to-all neuronal networks. The constituent neurons implemented the Mahowald-Douglas model^30^ which transposes the conductances of ion channels into transistor conductances to translate the Hodgkin-Huxley model^34^ to very large scale integrated (VLSI) technology. We interconnected these neurons with mutually inhibitory synapses based on established VLSI circuit design^31^. These synapses have three gate biases which we set independently or in combination to delay the onset of the postsynaptic current, change the rise and decay time of the postsynaptic current, and vary the synaptic conductance (Supplementary Methods I,II). Accordingly, individual synapses have a tuneable inhibition delay *d* which we vary from 20 *μ*s to model the latency time of neurotransmitter release^35,36^, to 800 *μ*s to model the transmission line delay of inhibitory signals as they diffuse along the dendrites towards to the axon hillock of dendrite projecting interneurons^37^. These inhibition delays are chosen to match the transit time of action potentials across the 200 *μ*m–700 *μ*m long dendrites of somatostatin interneurons^37^ at an average speed of 1–100 m/s (Fig. 1(a)). The decay (resp. rise) time of the postsynaptic current was set by the undocking (resp. docking) time of neurotransmitters on neuroreceptors (GABA), *τ*_*u*_ (resp. *τ*_*d*_). *τ*_*u*_ was tuned over 0–8 ms, a range comparable to the period of neuron oscillations: 5–20 ms^38^ (Fig. 1(b)).

### Synaptic kinetics of the half-center oscillator

We began to study the emergence of synchronization by probing the synchronization phase diagram of a pair of mutually inhibitory neurons as a function of synaptic kinetics in all connections (*d*, *τ*_*u*_) and current stimulation applied to all neurons (*I*_*stim*_). When inhibition delay is small ($d < 150\,\mu$s), three modes of synchronized oscillations are observed as *I*_*stim*_ increases (Fig. 1(c)). Above the depolarization threshold (*I*_*th*_ = 8 *μ*A), neurons oscillate out-of-phase (antiphasic synchronization). They suddenly lock in phase (phasic synchronization) at *I*_*s*_ = 14 *μ*A. Higher current stimulation (${I}_{stim} > {I}_{s}$) increases the frequency of neuron oscillations and makes inhibition increasingly tonic. As a result neurons decouple gradually. This loose coupling regime is characterized by higher order phase locking where a neuron entrain the other at a frequency which is a rational multiple of its own (Fig. 1(c)).

Longer inhibition delays ($d > 150\,\mu$s) broaden the synchronization current *I*_*s*_ to a window of finite width [*I*_*L*_, *I*_*H*_] (Fig. 1(d)) which increases and eventually diverges at $d > 300\,\mu$s. The observation of phasic synchronization at longer inhibition delay concurs with similar results obtained by Van Vreeswijk *et al*.^11^ when the synaptic response time becomes slower. Antiphasic, phasic, and loose coupling regimes form 3 domains in the *d* − *I*_*stim*_ phase diagram of Fig. 1(e) showing that phasic synchronization may be induced either by delaying inhibition or by applying a stimulation current close to *I*_*s*_. Delayed inhibition gives each neuron in the pair the time to depolarize prior to receiving inhibition from its partner. This condition is necessary but not sufficient to explain phasic synchronization. Inhibition delay also decreases the slope of the phase response curve of the post-synaptic neuron near the origin (Supplementary Methods II). This reduces the phase correction that mutual inhibition applies to the early and late firing neurons which has the effect of stabilizing synchronous oscillations.

For shorter inhibition delays ($d < 150\,\mu$s), the synchronization current (*I*_*s*_) and frequency (*f*_*s*_) decrease when *τ*_*u*_ increases. This dependency is well explained by calculating the frequency of phase synchronized oscillations *f*_*p*_ (Supplementary Discussion I) and its intercept with the excitatory response curve of a neuron (Fig. 1(d)). We find ${f}_{s}\sim {\tau }_{u}^{-\mathrm{1/3}}$ (Fig. 1(f)). This result concurs with the onset of *γ*-oscillations shifting to lower frequency (current stimulation) following pharmacological manipulations that increase the recovery time of the postsynaptic current^7,8^.

### 3-cell mutually inhibitory network

**Figure 2:**
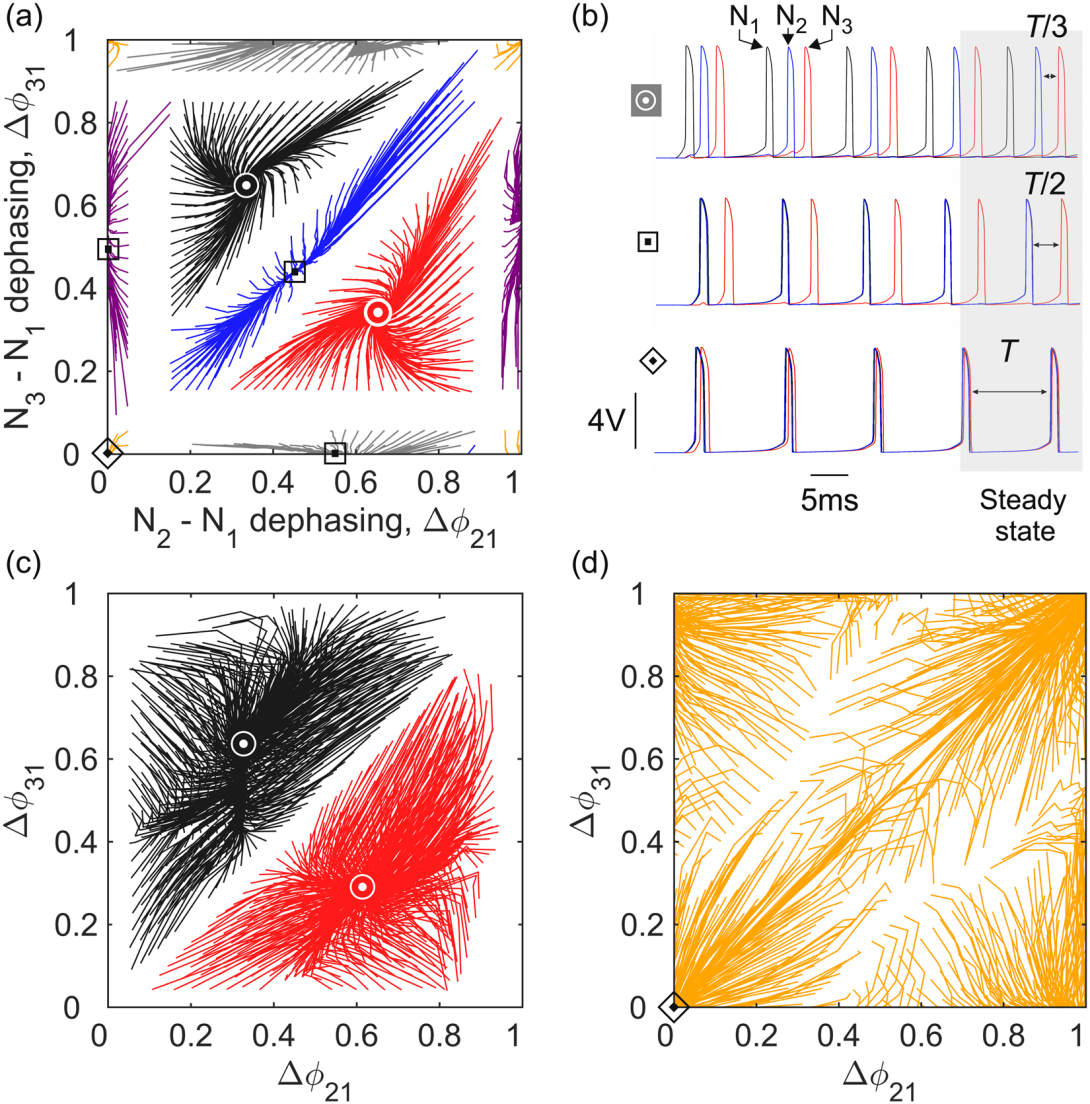
Phase portraits of 3-neuron inhibitory networks. (**a**) Experimental phase portrait of a three neuron network coupled via mutually inhibitory synapses. Antiphasic attractors (circle symbols), partially synchronized attractors (square symbols) and phasic attractor (diamond symbol) are the 6 limit cycle oscillations of the network. State trajectories (full lines) emanate from initial states evenly distributed over the entire phase space. Neuron dephasings ΔΦ_*i*1_ were normalised by the cycle period *T*. Reciprocal inhibition was balanced ${g}_{ij}\approx {g}_{ji}=2\,\,\mu$S with *i*, *j* = 1, 2, 3. (**b**) Transient neuron oscillations showing convergence towards the antiphasic attractor (ISI = *T*/3), the partially synchronized attractor (ISI = *T*/2), and the phasic attractor (ISI = *T*). (**c**) Phase portrait of a 3-neuron network interconnected with mutually inhibitory gap junctions showing antiphasic attractors only (circle symbols). ${g}_{ij}\approx {g}_{ji}=45\,\,\mu$S. (**d**) If mutually excitatory gap junctions are used instead, a single phasic attractor is observed (diamond symbol). *Parameters*: (**a**,**b**) *I*_*stim*_ = 25 *μ*A, *T* = 18 ms, *I*_*th*_ = 8 *μ*A, ${g}_{ij}^{(s)}=2\,\mu$S, *τ*_*u*_ = 1.5 ms, *τ*_*d*_ = 1.5 ms, *d* = 300 *μ*s; (**c**,**d**) *I*_*stim*_ = 50 *μ*A, *I*_*th*_ = 86 *μ*A.

Larger inhibitory networks ($N\ge 3$) generally have chaotic dynamics which makes network oscillations highly dependent on the timings of current stimuli. We defined the state of the system using the phase lags of individual neurons relative to a reference (neuron 1) and obtained the state trajectories by measuring the temporal evolution of these phase lags $\{{{\rm{\Delta }}{\rm{\Phi }}}_{i1}^{(p)}\}$, *i* = 2, 3 ...*N* over consecutive periods *p* = 1–50. The phase lag map of a 3-neuron network with 300 *μ*s inhibition delay shows state trajectories converging towards 6 point attractors (Fig. 2(a)). These attractors are sub-divided into 3 categories according to the duration of their interspike intervals (ISI): *T*/3, *T*/2 and *T* where *T* is the period of synchronized oscillations (Fig. 2(b)). Two attractors (circle symbols) correspond to three neurons discharging in the clockwise and anticlockwise sequences, 1 → 2 → 3 and 1 → 3 → 2 (ISI = *T*/3). Three attractors (square symbols) correspond to 3 modes of partially synchronized oscillations including the sequence $$1\to \begin{array}{c}2\\ 3\end{array}$$ and its 2 permutations (ISI = *T*/2). The single coherent attractor (diamond symbol) corresponds to all 3 neurons discharging in phase (ISI = *T*). The 3-neuron map shows the basins of attraction becoming smaller as oscillations become more coherent. This demonstrates the greater fragility of coherent states relative to the oscillations of sequentially discharging neurons. We find that for $d > 300\,\mu$s, coherent and partially coherent oscillations become stable over the entire range of current stimulation. If $d < 150\,\mu$s however, the network only supports the oscillations of sequentially discharging neurons, as we shall see below. We find that substituting non-delayed inhibitory synapses (*d* = 0) with gap junctions^29^ produces qualitatively similar phase portraits in that they only support sequentially discharging neurons (Fig. 2(c)). For completeness, we also considered gap junctions between excitatory neurons. We find that the excitatory network hosts a single state of collective oscillations (Fig. 2(d)). This expected result validates the correct operation of our analogue network. Returning to the 3-neuron network connected by non-delayed inhibitory synapses, and varying current stimulation applied to all neurons, we find that partially coherent oscillations vanish except in a very narrow range of current stimulation centered on *I*_*s*_ - as in the neuron pair.

**Figure 3:**
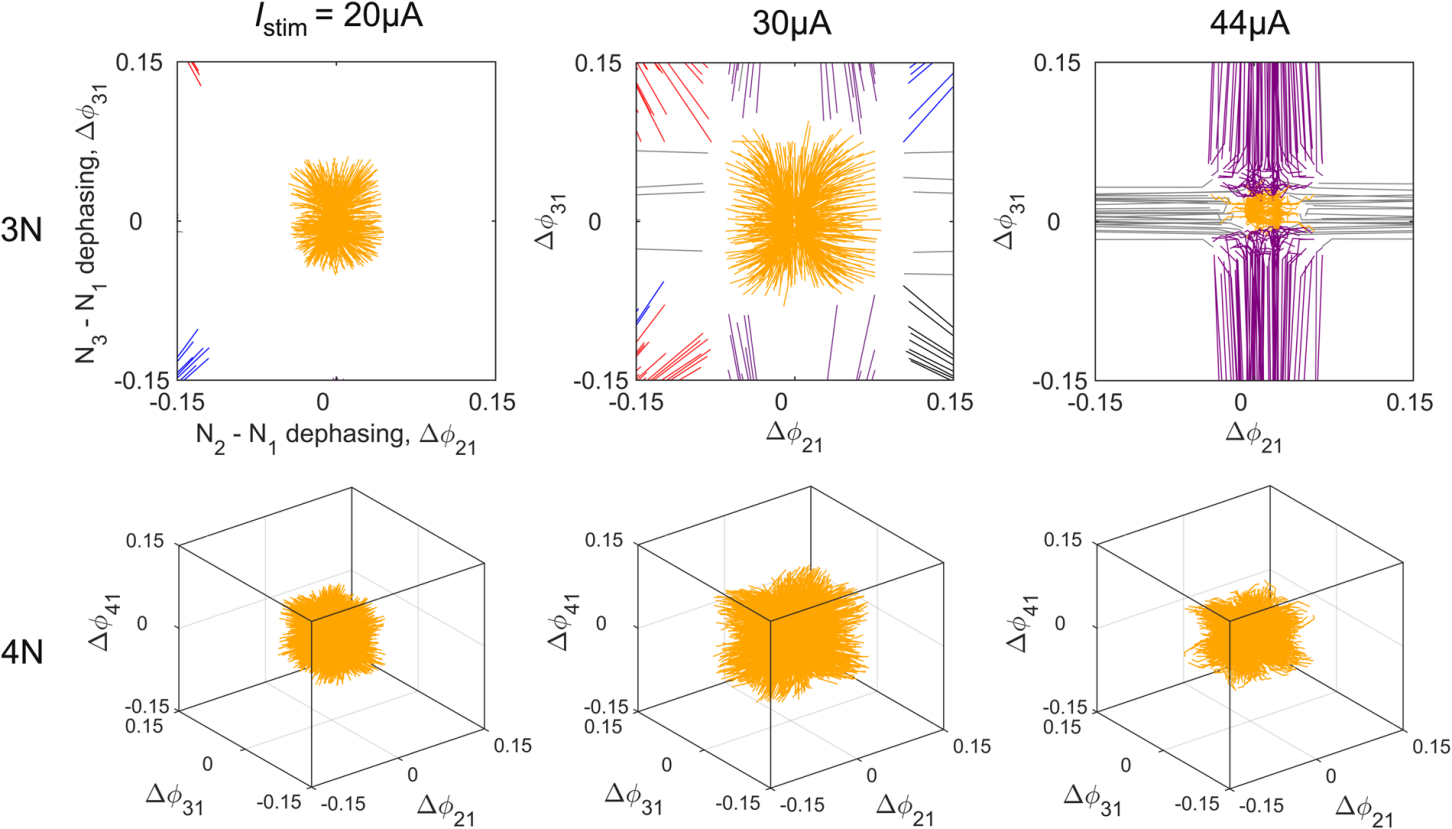
Current dependence of the coherent attractor. Phase lag maps of the 3-neuron and 4-neuron inhibitory networks measured in the vicinity of the coherent attractor (yellow basin) at three levels of current stimulation: *I*_*stim*_ = 20 *μ*A, 30 *μ*A and 44 *μ*A. Vicinal basins of partially synchronized oscillations (grey, purple and blue trajectories) and antiphasic oscillations (red and black trajectories). The volume of the coherent basin passes through a maximum at ${I}_{s}\approx 30\,\mu$A. *Parameters*: *d* = 350 *μ*A, *τ*_*u*_ = 1.5 ms.

In the 3-neuron and 4-neuron networks, the synchronization current *I*_*s*_ is the current that maximises the size of the coherent basin of attraction and stabilizes the coherent attractor with respect to noise (Fig. 3). For long inhibition delays (*d* = 350 *μ*s), the network supports coherent oscillations over the entire range of current stimulation. When $d < 150\,\mu$s, coherent oscillations only form in a narrow range of current stimulation about *I*_*s*_. These observations generalize the *d* − *I*_*stim*_ phase diagram of Fig. 1(e) to larger networks and demonstrate that synchronization may be achieved either through increases in inhibition delay or current stimulation.

### Emergence of synchronization in all-to-all inhibitory networks

We next demonstrate the emergence of synchronization in larger networks (*N* = 3, 4, 5) and the critical importance of inhibition delay in stabilizing locally coherent oscillations. The maximum number of attractors in a *N*-neuron network was calculated by counting the number of cyclically invariant discharge patterns allowing partial synchronization (Supplementary Discussion II). We find that the maximum network capacity increases as *T*_3_ = 6, *T*_4_ = 26, *T*_5_ = 150, *T*_6_ = 1082, … ${T}_{N}\sim (N-1)!/{(\mathrm{ln}2)}^{N}$^39^. The minimum capacity, allowing sequential discharges only, is *L*_*N*_ = (*N* − 1)!

**Figure 4:**
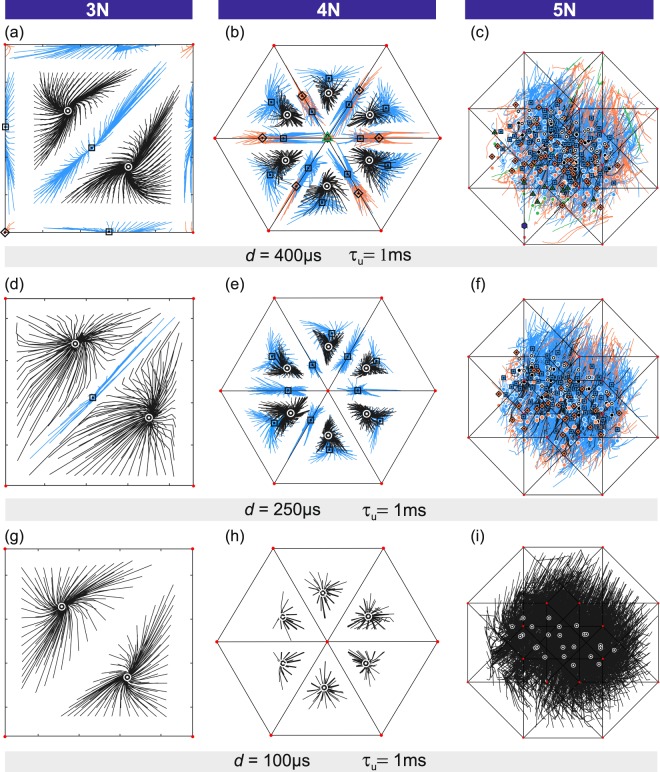
Emergence of synchronization in small inhibitory networks and its dependence on inhibition delay. Phase lag maps of the 3, 4 and 5-neuron networks measured at inhibition delays (**a**–**c**) *d* = 400 *μ*s, (**d**–**f**) *d* = 250 *μ*s and (**g**–**i**) *d* = 100 *μ*s while keeping constant both the decay time of the postsynaptic current: *τ*_*u*_ = 1.5 ms and the inhibition peak current: −13.8 *μ*A. The (*N* − 1)-dimensional phase space (straight lines) and the state trajectories within it (full lines) were projected orthographically. State trajectories converge towards point attractors classified according to the duration of their ISIs: *T*/*N* (black lines, circle attractors), *T*/(*N* − 1) (blue lines, square attractors), *T*/(*N* − 2) (orange lines, diamond attractors), *T*/(*N* − 3) (green lines, triangular attractors), *T*/(*N* − 4) (purple lines, hexagonal attractor). The total number of attractors observed at inhibitory delay *d* = 400/250/100 *μ*s is 6/3/2 (*N* = 3), 26/17/6 (*N* = 4), 142/107/24 (*N* = 5), 1053/688/120 (*N* = 6).

Experimental results show that the capacity of an inhibitory network to encode information about its environment lies between *L*_*N*_ and *T*_*N*_, depending on inhibition delay (Fig. 4). Longer inhibition delays (*d* = 400 *μ*s) stabilize oscillations which range from purely phasic (Fig. 4: (a) diamond, (b) triangle, (c) hexagon) to purely sequential (Fig. 4(a–c) circles). In between, all intermediate states of partial synchronization are observed (Fig. 4(a–c)). For example, the 4-neuron map in Fig. 4(b) has 6 sequential attractors with 1 spike per ISI giving ISI occupancies (1, 1, 1, 1) (circle symbols), 12 partially synchronized attractors with ISI occupancies (2, 1, 1, 0) (square symbols), 4 + 3 partially synchronized attractors with (3, 1, 0, 0) and (2, 2, 0, 0) occupancies respectively (diamond symbols), and the coherent attractor (4, 0, 0, 0) (triangle symbol). Therefore the 4-neuron network hosts 26 attractors in total.

Intermediate inhibition delay (*d* = 250 *μ*s) suppresses coherent oscillations (Fig. 4(d–f)). In the 4-neuron network, the coherent attractor (ISI = *T*) and the partially coherent attractors (ISI = *T*/2) have vanished while those with ISI = *T*/3 (square symbols) and *T*/4 (circle symbols) remain. The partially coherent attractors which survive exhibit a reduced basin size (Fig. 4(d,f)).

When inhibition delay is reduced further (*d* = 100 *μ*s), the only attractors left are sequential oscillations (Fig. 4(g–i)). The network capacity then scales as: 2 (*N* = 3), 6 (*N* = 4), 24 (*N* = 5) which matches the *L*_*N*_ sequence above. These results demonstrate that, provided the inhibition delay is sufficiently large, the number of attractors increases according to sequence *T*_*N*_. For this, the inhibition delay needs to be at least 1/3 of the duration of the action potential ($d > W\mathrm{/3}$). The network capacity was found to be less sensitive to neurotransmitter kinetics. Increasing *τ*_*u*_ from 1.5 ms to 3.5 ms marginally increased the number of attractors. No further change was observed beyond ${\tau }_{u} > 3.5$ ms.

**Figure 5:**
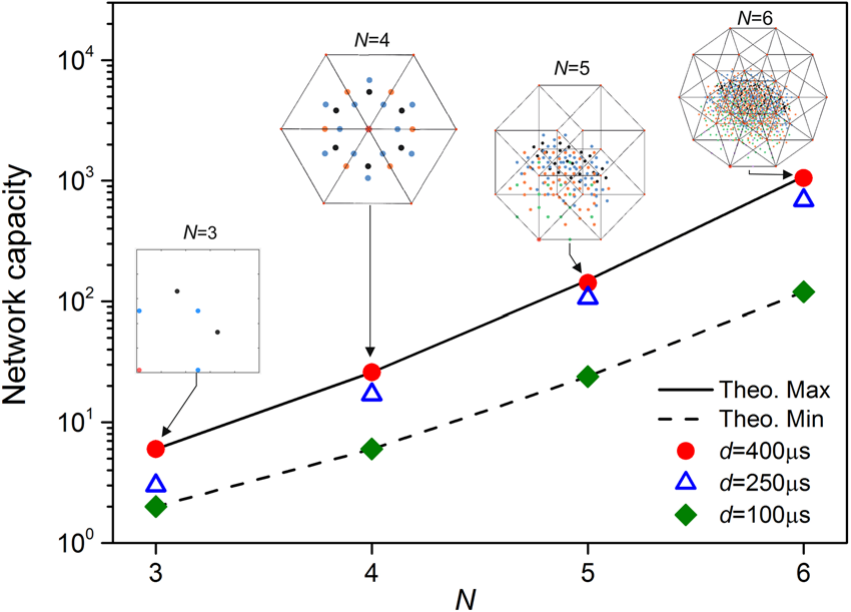
Scaling of network capacity with network size. Total number of attractors observed in the 3-neuron to 6-neuron networks at three different values of the inhibition delay: *d* = 400 *μ*s (red dots), 250 *μ*s (blue triangles), 100 *μ*s (green diamonds). At intermediate delay (250 *μ*s), the network capacity lies between the upper theoretical boundary *T*_*N*_ (solid line) and the lower boundary *L*_*N*_ (dashed line). *Inset*: Orthographic projections of point attractors which are distinguished by the number of ISIs per cycle: ISI = *T*/*N* (black dots), *T*/(*N* − 1) (blue dots), *T*/(*N* − 2) (orange dots), *T*/(*N* − 3) (green dots), *T*/(*N* − 4) (purple dot).

Figure 5 shows how the capacity of experimental networks scales with network size. At small inhibition delay (*d* = 100 *μ*s), the experimentally observed capacity is minimum and follows sequence *L*_*N*_. At longer inhibition delay (*d* = 400 *μ*s), one observes that the maximum number of attractors increases according to sequence *T*_*N*_. At intermediate delays, the network supports partially synchronized oscillations with low coherence which includes all oscillations exhibiting the smaller ISIs. Hence the network capacity lies between *L*_*N*_ and *T*_*N*_. One concludes that longer inhibition delays ($d > 300\,\mu$ s) boost the capacity to encode stimuli by a factor ${T}_{N}/{L}_{N}={(\mathrm{ln}\mathrm{2)}}^{-N}$. With a maximum capacity of (*N* − 1)!/(ln2)^*N*^ delayed inhibitory networks achieve a storage density which far exceeds winnerless networks $\sim (N-1)!$^24^ and Hopfield networks $\sim 0.14N$^40^. By achieving the maximum theoretical capacity, our *in-silico* networks demonstrate scalable associative memories with unprecedented memory density.

## Discussion

Our results suggest that inhibitory networks may synchronize via two mechanisms that exploit the distinct neurophysiological properties of fast-spiking interneurons^36^ and the inhibition delay introduced by dendrite projecting synapses^37^. This study considers the primary effect of dendrite targeting synapses to be the introduction of a transmission line delay because the network frequency covers a very narrow range set by the constant step amplitude of current stimuli. The complex spectral response of dendrites is however known to be important and would need to be considered if the amplitude of current stimulation was varied. Dendrite projecting somatostatin interneurons introduce transmission line delays of the order of 0 – 800 *μ*s by projecting synapses on the 200–700 *μ*m long dendrites of the mammalian visual cortex^37^. Transmission line delays of this magnitude postpone the onset of inhibition sufficiently to stabilize the coherent oscillations of inhibitory neurons (Fig. 4(a–c)). The anatomical properties of somatostatin neurons would thus warrant robust phasic synchronization which is weakly dependent on current stimulation or postsynaptic kinetics but is strongly dependent on the timings of stimulation. This result is consistent with the rapid attenuation of visually induced *γ*-oscillations observed when visual stimuli become uncorrelated^6^. The coherent attractor is unique and its basin occupies a very small volume of phase space (triangle symbol, Fig. 4). As a result, the state of collective synchronization is the least robust of all states with respect to noise and structural inhomogeneity. In contrast, the bulk of the phase space is filled with partially coherent attractors whose proportion increases very rapidly according to 1 − (ln2)^*N*^ as the network size increases. Using this expression, one calculates that partially coherent attractors form $> \mathrm{98.7 \% }$ of all attractors for the typical neuronal population, $N > 12$, excited during optogenetic experiments^6^. Besides being more numerous, partially coherent states also have wider basins which offer protection from decoherence by noise and structural heterogeneities (Fig. 4(a–c)). Accordingly, partially coherent states are the most thermodynamically stable with respect to coherent and sequential states and are the most likely to support synchronized electrical activity in the noisy environment of real cortical networks. Within partially coherent states, however, the neurons which oscillate in phase may distribute differently over the volume of the network. A subset of *L* neurons ($L < N$) may oscillate in phase at different locations of the network, producing spatially homogeneous firing akin to the fully synchronized state. Two partially coherent states with identical *L*-number differ through the permutations of stimuli. The equivalence of these states is demonstrated by the six-fold symmetry of phase maps of the 4-neuron network (Fig. 4(b)).

Our results suggest that spatially homogeneous firing within partially coherent states may be promoted by local repulsion through gap junctions^41^. These junctions are known to predominantly couple neighbouring inhibitory cells of the same population^42,43^. As we have seen in Figs 2(c) and 4(g), gap junctions and fast inhibitory synapses share the property of supporting sequential neuronal oscillations. Electrical synapses thus have a destabilizing effect on local neural synchronization as reported in earlier numerical simulations^21,23,44^. At the same time, Fig. 4 show that transmission line delays promotes synchrony. An inhibitory network can thus achieve a homogeneous distribution of phasic neurons^45^ by breaking local coherence using gap junctions. Homogeneous firing is established from the long range attraction of delayed inhibition and the short range repulsion of electrical synapses. Note that many physical systems achieve long range order through short range repulsion. For example, the Wigner crystal arises from Coulomb repulsion between electrons^46^ and vortex-to-vortex repulsion is responsible for the Abrikosov lattice in type II superconductors^47^. The effect of introducing heterogeneity in the network is seen in Fig. 4(a–c) where residual imbalance in network conductance breaks the symmetry of phase lag maps. Introducing a range of inhibition delays or mixing gap junctions with chemical synapses would similarly increase the volume of some basins - those associated with spatially homogeneous firing - to the detriment of others^26^.

In contrast to somatostatin neurons, the wiring of parvalbumin neurons introduces delays which are too short to warrant automatic synchronization. Instead parvalbumin neurons may achieve synchronization through high frequency entrainment. This corresponds to the current induced synchronization which we observe at small *d* (Fig. 1(c,e)). Because frequency *f*_*s*_ is dependent on neurotransmitter kinetics (Fig. 1(f)), this synchronization mechanism allows the onset of synchronized oscillations to be tuned using pharmacological manipulations targeting GABA receptors^4,5,7,9,48^.

Our study leads us to propose that local cortical circuits may have adapted to exploit the robustness of synchronization by delayed inhibition versus the tunability of synchronization by fast-spiking interneurons (Fig. 1(e)). These synchronization mechanisms suggest strategies to reduce pathological cortical oscillations which include: inactivating dendrite targeting synapses, blocking GABA_*B*_ receptors to accelerate the recovery of the postsynaptic potential, and applying visual stimuli lacking spatial coherence at frequencies in the *γ* band. This study has focussed on purely inhibitory networks (ING) which have intrinsically chaotic dynamics. The consideration of excitatory neurons and feed-forward processes within the pyramidal-interneuron-gamma (PING) mechanism invokes regular dynamics which has been treated elsewhere^8^.

## Methods

### Electronic models

We synthesized two VLSI networks interconnecting 6 Mahowald-Douglas neurons^30^ with either inhibitory synapses or gap junctions (Supplementary Methods I). VLSI neurons modelled the dependence of the membrane voltage *V* on current stimulus *I*_*stim*_ using the analogue electrical equivalent circuit of the neuron membrane. Its equation was $C\dot{V}={g}_{Na}({E}_{Na}-V)+{g}_{K}({E}_{K}-V)+{g}_{L}V+{I}_{stim}$ where *E*_*Na*_ and *E*_*K*_ are the sodium and potassium reversal potentials and *C* is the membrane capacitance. The sodium and potassium conductances, *g*_*Na*_ and *g*_*K*_, are modelled by the transconductances of *p*− and *n*− type field effect transistors respectively^30^. The gate variables *m*, *h* and *n* of the Hodgkin-Huxley model are represented in the analogue circuit by currents *ι* which are either activated or inactivated according to: $\iota ({V}_{\tau ,x})={\iota }_{max}\{1+\,\tanh \,[({V}_{\tau ,x}-{V}_{x})/d{V}_{x}]\}/2$ where *x* ≡ {*m*, *h*, *n*}, *V*_*x*_ is the threshold voltage of each ion gate, and *dV*_*x*_ is the width of the transition from the closed to the open state of that gate. The *V*_*τ*,*x*_ variables follow a first order dynamics ${\dot{V}}_{\tau ,x}=(V-{V}_{\tau ,x})/\tau x$ which describes the recovery of each gate variable and is characterized by recovery time *τ*_*x*_^29^.

Chemical synapses were implemented using a differential pair integrator^31^ (Supplementary Methods II). As our transistors functioned with above threshold currents as opposed to below threshold^31^, the postsynaptic current was approximately given by *I*_*post*_(*t*) = *gS*(*t*)(*V*_*post*_(*t*) − *V*_*rev*_) where *V*_*rev*_ = 7 V was the reversal potential, *V*_*post*_(*t*) the membrane voltage of the postsynaptic neuron, *g* the maximum conductance and *S*(*t*) was the fraction of docked neurotransmitters at time *t*. The neurotransmitter docking rate was given by: $\dot{S}(t)=[{S}_{\infty }({V}_{pre}(t))-S(t)]/{\tau }_{u}$ with ${S}_{\infty }(V)=0.5\{1+\,\tanh \,[(V-{V}_{th})/d{V}_{syn}]\}$. The empirical inhibition delay *d*, decay time *τ*_*u*_ and synaptic conductance *g* were controlled by 3 gate voltage parameters: *V*_*th*_, *V*_*W*_ and *V*_*τ*_ in the circuit (Supplementary Methods II). The synaptic conductance varied in the range *g* = 1–3 *μ*s.

We implemented gap junctions electronically using a differential transconductance amplifier to model electrical coupling between GABAergic-like interneurons^41^. Their current-voltage transfer characteristics has been measured by Zhao and Nogaret^29^. The gap junction current varies linearly as ${I}_{post}=g^{\prime} ({V}_{post}(t)-{V}_{pre}(t))$ near the balance point of the pre-synaptic and post synaptic membrane potentials^41^. The transconductance $g^{\prime}$ is tuneable in the range 24 *μ*s $< g^{\prime}  < 45\,\mu$S using the gate bias *V*_*M*_ of the current source transistor (Fig. S7). Away from the balance point, saturation effects reduce the rate of current injection^29^. We were able to change the sign of the injected current by swapping the voltage inputs and in this way obtain either an inhibitory or an excitatory link (Fig. 2(d)).

Circuits were built from VLSI current mirrors (ALD1116, ALD117). The depolarization threshold of neurons was adjusted to match the range of synaptic currents. This was done by adjusting the leakage conductance of the neuron membrane. The current thresholds were *I*_*th*_ = 8 *μ*A (synaptic coupling) and 86 *μ*A (gap junction coupling). The duration of an action potential was *W* = 1 ms.

### Data acquisition and analysis

Individual neurons were stimulated by timed current steps of constant amplitude *I*_*stim*_. These stimuli were generated by the analogue outputs of two DAQ cards (NI PCI6259) and a bank of 6 voltage-to-current converters. Labview code was written to vary the timings of current stimuli in a systematic manner so that initial conditions meshed the (*N* − 1)-dimensional phase space with a grid size of *T*/20. The Labview/DAQ card recorded the membrane voltage time series of individual neurons during each current protocol. The sampling frequency was 20 kHz. Between the end of one protocol and the beginning of the next, a 200 ms long time window was inserted during which no stimulation was applied to let the system return to its steady state.

The dephasings of voltage peaks $({{\rm{\Delta }}{\rm{\Phi }}}_{21}^{(p)},{{\rm{\Delta }}{\rm{\Phi }}}_{31}^{(p)},\,\mathrm{...}{{\rm{\Delta }}{\rm{\Phi }}}_{N1}^{(p)})$ were calculated in each oscillation period *p* = 1–50. The phase shifts of individual neurons were calculated as ${{\rm{\Delta }}{\rm{\Phi }}}_{i1}^{(p)}=({t}_{i}^{(p)}-{t}_{1}^{(p)})/T$ using a Matlab programme which extracted the timings of voltage peaks of neuron *i* and neuron 1 in each oscillation period. The state trajectories Δ**Φ**^(*p*)^ were projected orthographically in the Coxeter plane of the (*N* − 1)-dimensional hypercube (*N* = 3, 4, 5) using projection matrices:

1 $${\hat{{\boldsymbol{P}}}}_{4N}=(\begin{array}{ccc}-\sqrt{2}\,\cos \,{\theta }_{4} & \sqrt{2}\,\sin \,{\theta }_{4} & 1\\ \sqrt{2}\,\sin \,{\theta }_{4} & -\sqrt{2}\,\cos \,{\theta }_{4} & 1\end{array}),$$

where *θ*_4_ = *π*/12, and:

2 $${\hat{{\boldsymbol{P}}}}_{5N}=(\begin{array}{cccc}1 & \cos \,{\theta }_{5} & 0 & -\,\cos \,{\theta }_{5}\\ 0 & \sin \,{\theta }_{5} & 1 & \sin \,{\theta }_{5}\end{array}),$$

where *θ*_5_ = *π*/4. The state trajectories pertaining to the same basin were regrouped using Matlab code which calculated the coordinates of experimental attractors and their total number.

## Electronic supplementary material

Supplementary information
