# Supplementary material for: Dual Mechanism for the Emergence of Synchronization in Inhibitory Neural Networks
Source: Sci Rep. 2018 Jul 30;8:11431. doi: 10.1038/s41598-018-29822-8 (PMC6065321; doi:10.1038/s41598-018-29822-8)
Supplement: Supplementary file 1 — Supplementary information [file 41598_2018_29822_MOESM1_ESM.pdf]

Dual Mechanism for the Emergence of Synchronization in Inhibitory Neural Networks

Ashok S. Chauhan, Joseph D. Taylor, Alain Nogaret
Department of Physics, University of Bath, Bath, BA2 7AY, UK

<u>Supplementary Methods I</u>: VLSI inhibitory networks

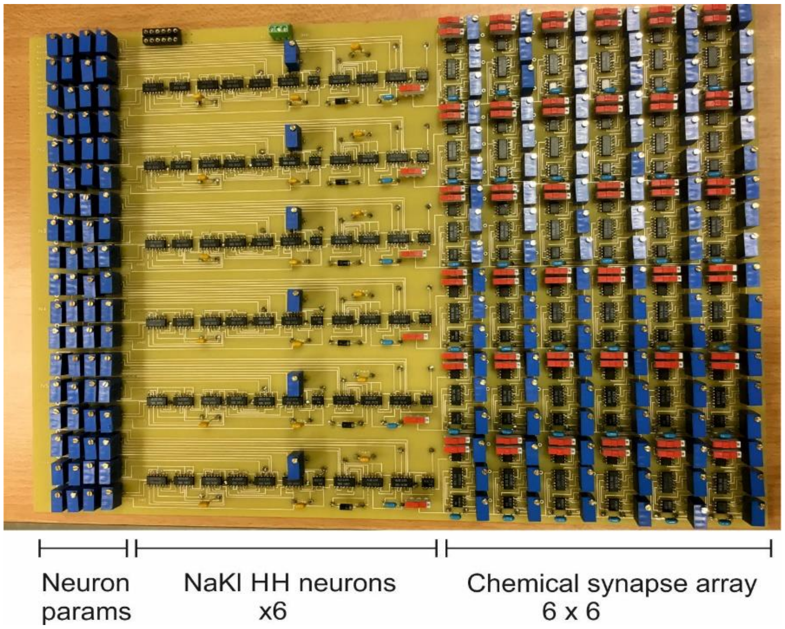

Figure S1: All-to-all mutually inhibitory network of 6 NaKl neurons and 36 synapses
The analogue network integrates of $6\times4+36=60$ coupled differential equations in real time. Time dependent current stimuli are injected in the top left DIL port. The same port is used to measure the time series membrane voltages. A second network was realized with gap junctions instead of chemical synapses. The number of neurons in the network was increased or decreased by setting synaptic conductances to zero.

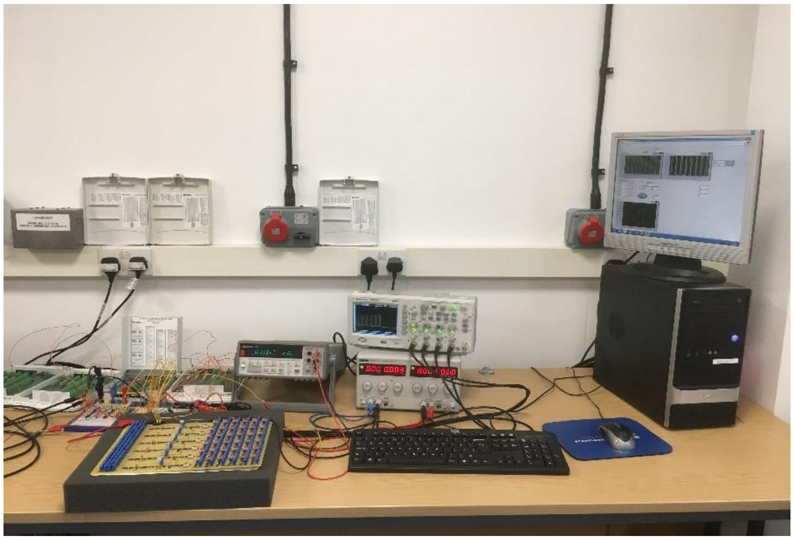

Figure S2: Data acquisition bench used to acquire the phase lag maps of analogue inhibitory networks
DAQ cards (NI6259 x 2 top left) inject timed currents stimuli into individual neurons and record the time series data of their membrane voltage. A Labview program controls the application of stimuli and records the membrane voltage.

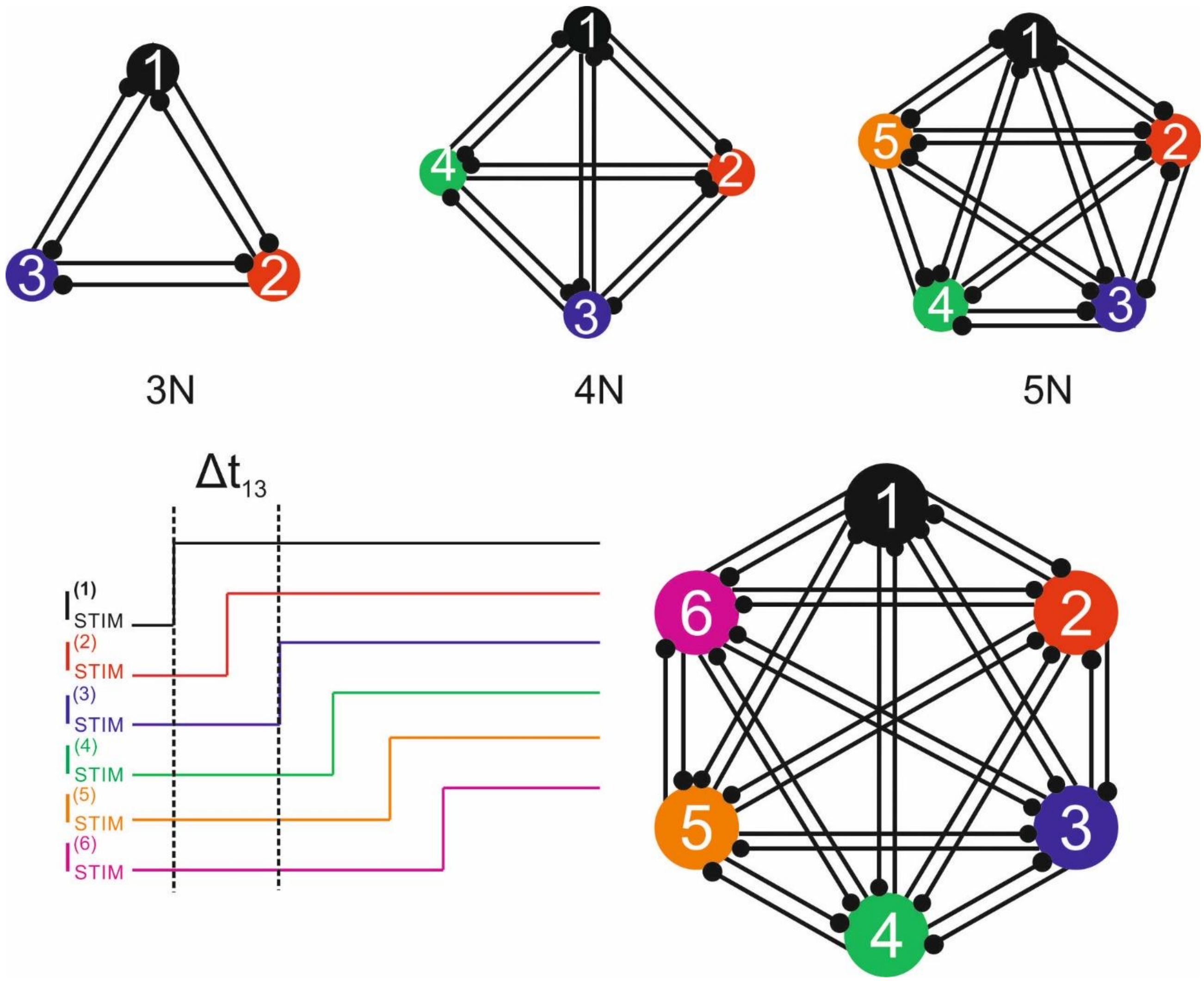

Figure S3: The all-to-all inhibitory networks implemented with 3, 4, 5 and 6 neurons
Current protocols applied to a network consist of current steps which are timed relative to the onset of stimulation of neuron 1. The timings are systematically varied over the period of oscillation of the membrane voltage.

Supplementary Methods II: VLSI synapses and gap junctions

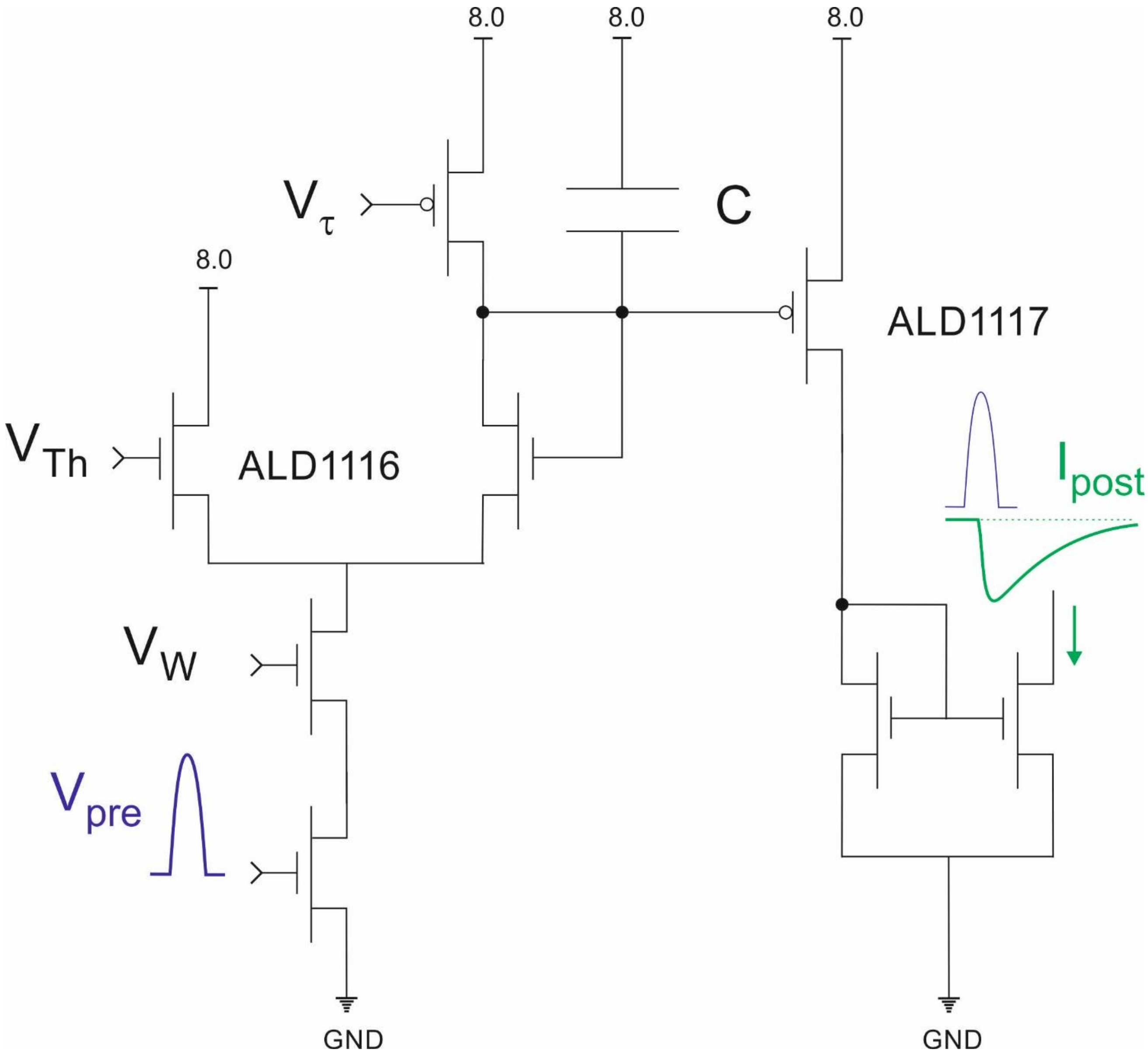

Figure S4: Inhibitory chemical synapse

The synapse is based on the differential pair integrator[1]. Presynaptic signals, V_{pre}, increase the conductance of the presynaptic MOSFET. The drain current of this MOSFET is modulated by V_W which sets the synapse weight. The V_{Th} MOSFET sets the knee voltage which determines the activation threshold of the synapse. The docking time (τ_d) and undocking time (τ_u) of neurotransmitters are determined by the charge discharge times of capacitor *C* through the V_τ MOSFET. Because the capacitor charges and discharges through different channels, the raise time of the postsynaptic current is a little faster than the decay time ($\tau_u > \tau_d$). τ_u and τ_d were tuned with V_τ. The *delayed synaptic response d* and the saturation current were tuned with parameters V_{Th} and V_W .

[1] C. Bartolozzi and G. Indiveri, *Neural Computation* **19**, 2581–2603 (2007)

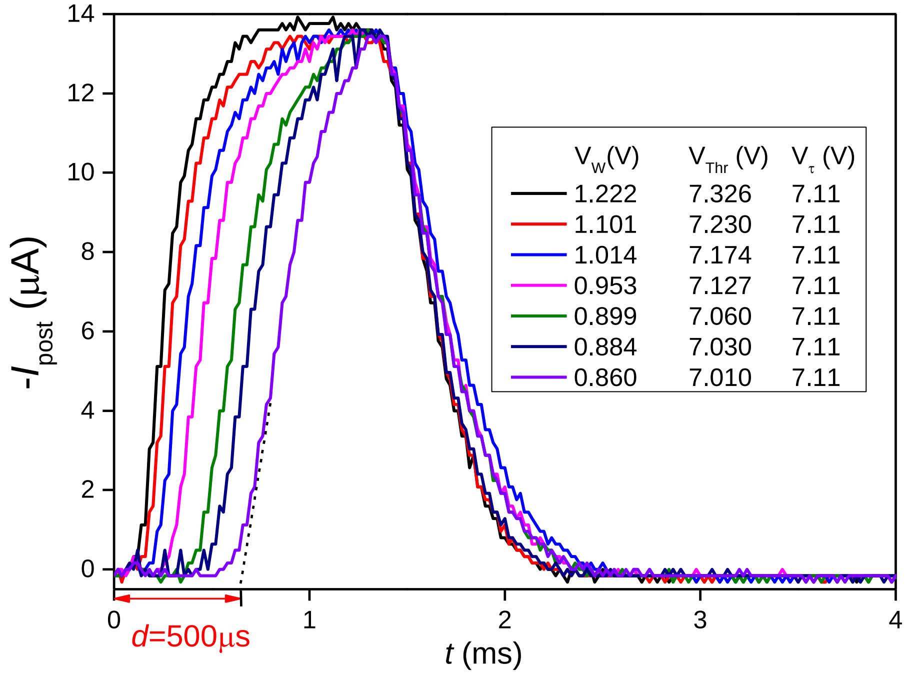

Figure S5: Controlling the inhibition delay *d* with synaptic parameters V_W and V_{Th}.

Inhibition delay was increased through the combined action of V_W which decreased the synaptic conductance and V_{Th} which decreased the input voltage threshold. The peak synaptic current was kept constant at $-13.8\mu A$.

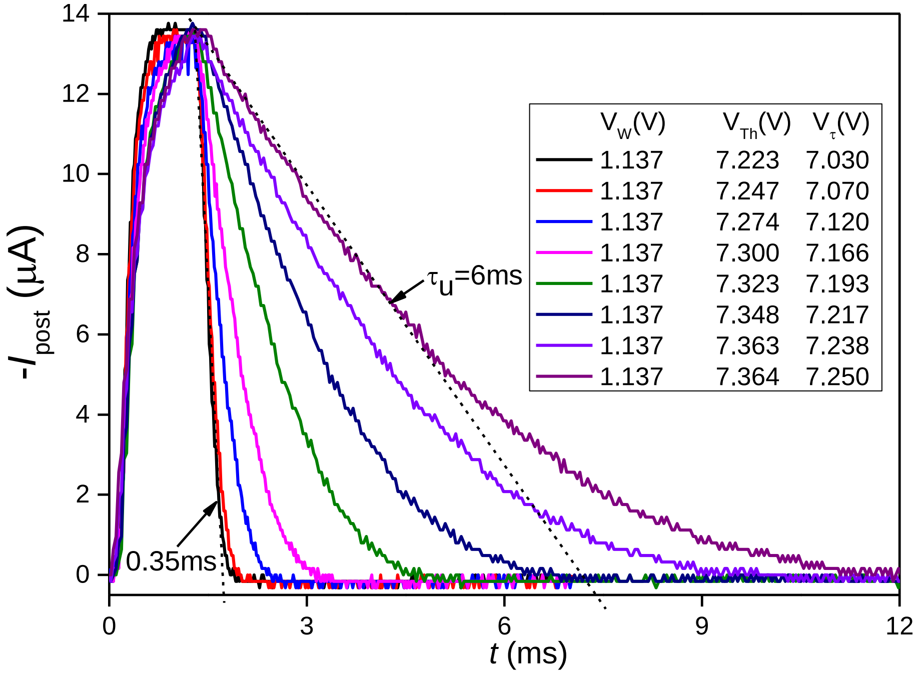

Figure S6: Controlling the decay of the postsynaptic current (neurotransmitter undocking time τ_u) with V_{Th} and V_τ

The neurotransmitter docking τ_d and undocking times τ_u were increased by increasing V_τ. This parameter also decreases the peak synaptic current, hence we increased V_{Th} to compensate and keep the peak current at -13.8μA.

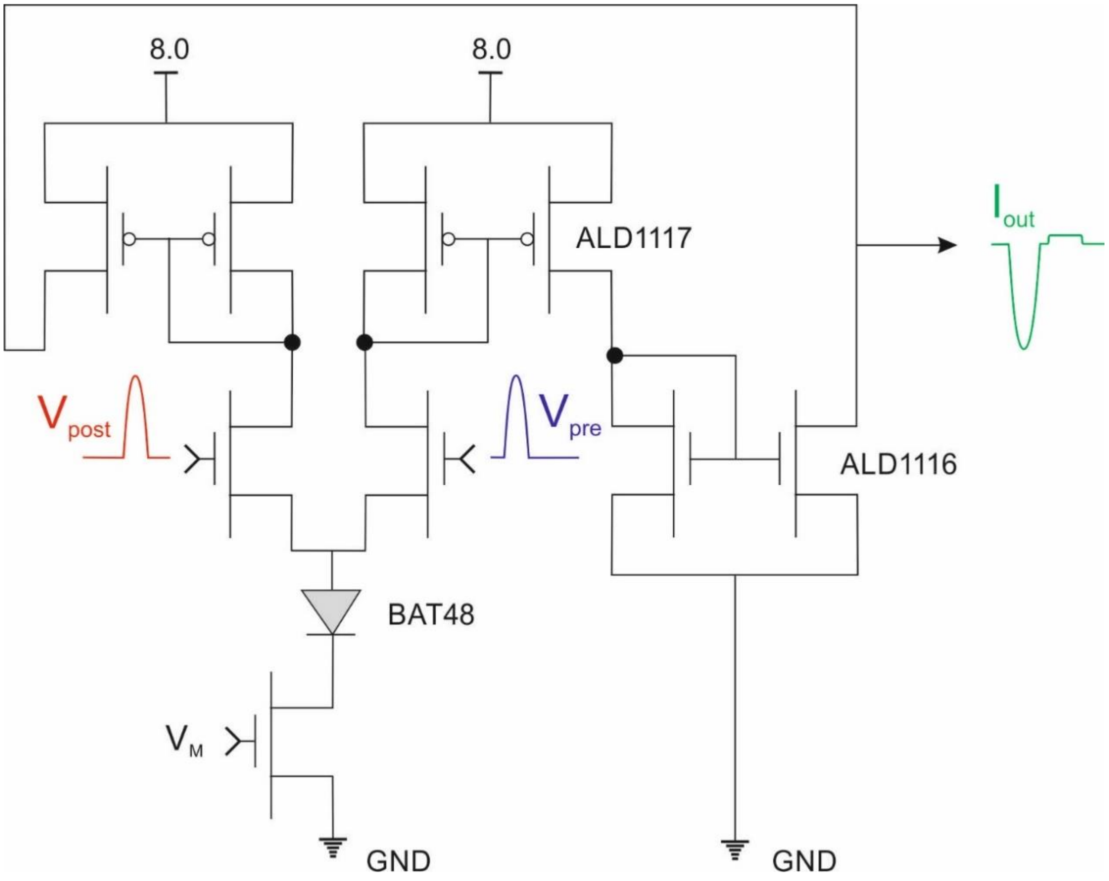

Figure S7: Inhibitory gap junction
The gap junction circuit is based on a transconductance differential amplifier giving $I_s = g(V_M)(V_{post} - V_{pre})$. The current saturates when the voltage difference of the pre- and post-synaptic neurons increases[2]. The positive rebound current induced when $V_{post} > V_{pre}$ was clipped by diode (BAT48). This rectification was needed to model the predominantly inhibitory current of fast spiking cells connected by electrical synapses[3]. The conductance of the gap junction was tuned with the gate voltage (V_M) of the current source transistor.

[2] L. Zhao and A. Nogaret, *Phys. Rev.* E **92**, 052910 (2015)
[3] M. Galarreta and S. Hestrin, *Science* **292**, 2295 (2001)

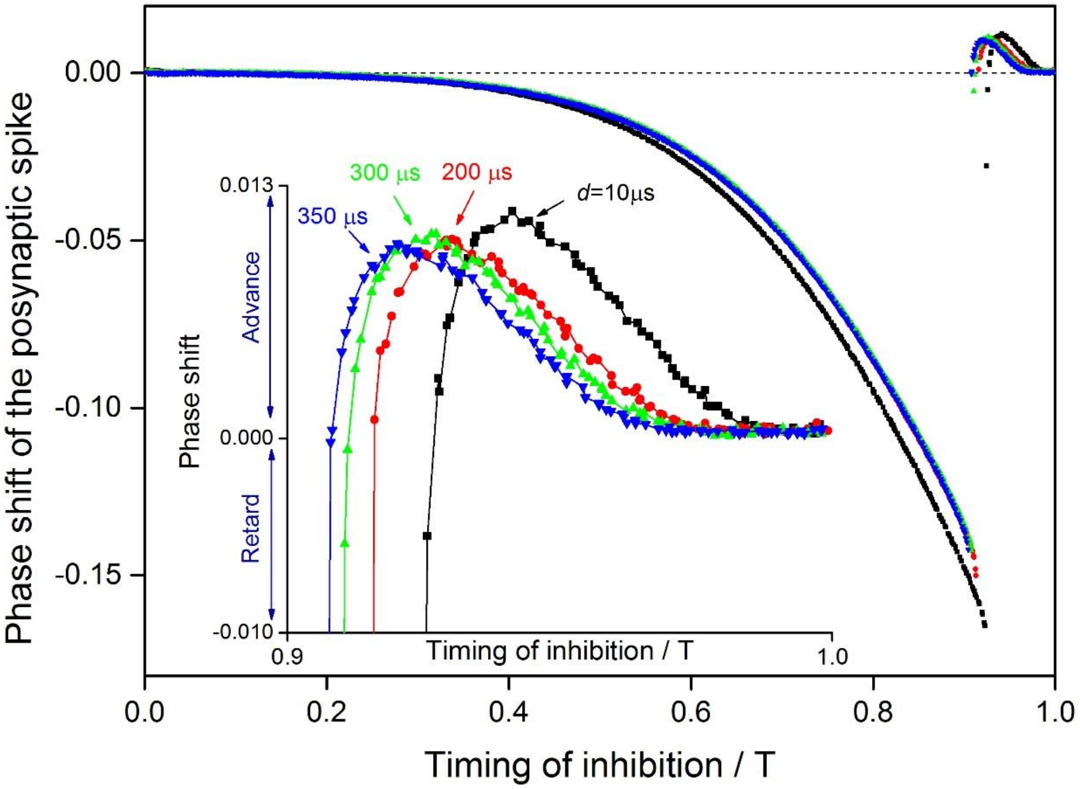

Figure S8: Phase response curve of the postsynaptic neuron and its dependence on inhibition delay d
The inset shows the effect of inhibition applied immediately before the postsynaptic spike. Inhibition delay decreases the slope of the phase response curve at $\Delta t = T$. This reduces the phase shift differential applied to early and late firing neurons in the inhibitory pair whose oscillations synchronize in phase rather than antiphase.

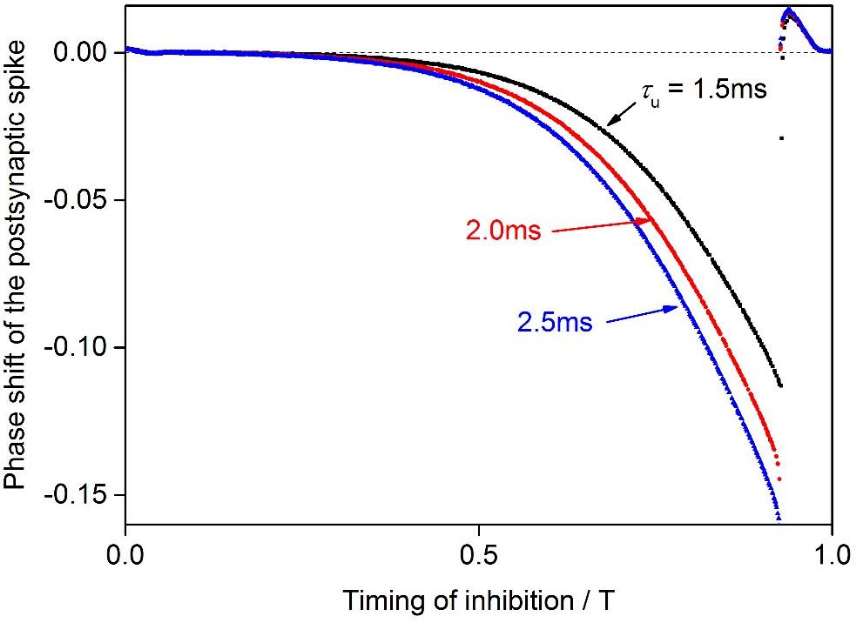

Figure S9: Phase response curve of the postsynaptic neuron and its dependence on τ_u
The negative phase shift arises from the postsynaptic spike being delayed by inhibition. Longer inhibition decay causes this shift to begin earlier in the cycle.

Supplementary Discussion I: Frequency of phasic oscillations of a pair of mutually inhibitory neurons

A delayed onset of inhibition is necessary for inhibitory neurons to synchronize in phase. This delay grants a finite time interval from the time a neuron begins to depolarize until it begins to receive inhibition from the other neuron [Figure S10(a)].

The frequency of phasic oscillations f_p [Fig. S10(b)] is calculated as a function of the following parameters:

- Tonic stimulation applied to each neuron, I_{stim}
- Current threshold: I_{th}
- Maximum postsynaptic current (all docking sites occupied): I_0
- Postsynaptic delay: d
- Neurotransmitter docking time: τ_d
- Neurotransmitter undocking time: τ_u
- Spike width: W

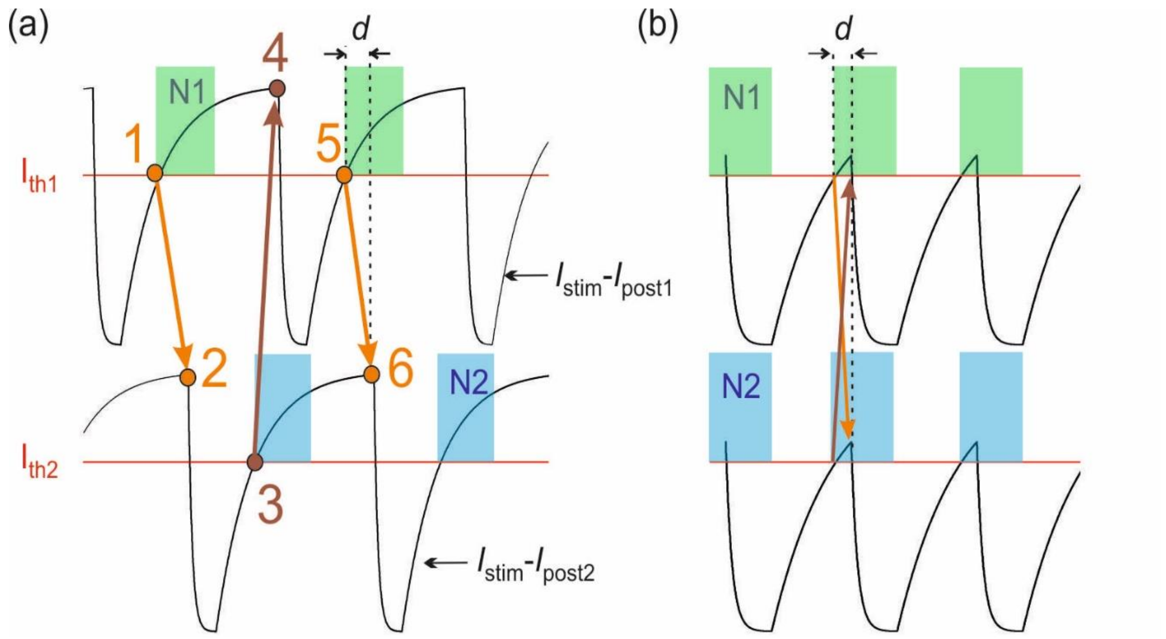

Figure S10: Time dependence of current stimulation during antiphasic and phasic synchronization
The net current injected into a neuron is the tonic stimulation minus the inhibition current: $I_{inj} = I_{stim} - I_{post}$.

(a) Antiphasic oscillations occur when $I_{stim} < I_s$

(1) current injection into N1 raises above the depolarization threshold causing N1 to depolarize. (2) N1 applies delayed inhibition to N2. Inhibition of N2 (I_{post2}) rises to a maximum at the end of the N1 spike then decreases until (3). At (3) N2 depolarizes. (4) N2 applies delayed inhibition to N1.

(b) Phasic oscillations occur when $I_{stim} \approx I_s$

The period of neuron oscillations now becomes comparable to the recovery time of inhibition. The delay of mutual inhibition (arrows) allows the neurons to depolarize simultaneously. Without this delay inhibition would block neuron depolarization.

The rise and decay of the postsynaptic current is exponential as follows:

$$I_{post}(t) = \begin{cases} (I_0 - I_{\min})\left(1-\exp\left(-\frac{t-d}{\tau_d}\right)\right) + I_0 & d \le t \le W \\ I_{\max}\exp\left(-\frac{t-W}{\tau_u}\right) & W \le t \le T+d \end{cases} \quad \text{(SI.1)}$$

Requiring the continuity of the current at $t = W$ and $t = T + d$ gives $I_{\min}$ and $I_{\max}$.
At $t = W$, $I_{post}(W) = I_{\max}$, the top equation in SI.1 gives:

$$I_{\max} = (I_0 - I_{\min})\left(1-\exp\left(-\frac{W-d}{\tau_d}\right)\right) + I_0 \quad \text{(SI.2)}$$

At $t = T + d$, $I_{post}(T+d) = I_{\min}$, the bottom equation gives:

$$I_{\min} = I_{\max}\exp\left(-\frac{T+d-W}{\tau_u}\right) \quad \text{(SI.3)}$$

Setting $x \equiv W - d$ and combining Eq. SI.2 ad SI.3 gives:

$$\begin{aligned} I_{\max} &= I_0 \frac{1-\exp\left(-x/\tau_d\right)}{1-\exp\left(-(T-x)/\tau_u - x/\tau_d\right)} \\ I_{\min} &= I_0 \exp\left(-(T-x)/\tau_u\right)\frac{1-\exp\left(-x/\tau_d\right)}{1-\exp\left(-(T-x)/\tau_u - x/\tau_d\right)} \end{aligned} \quad \text{(SI.4)}$$

Neurons depolarize at $t = 0$ when current injection crosses the neuron threshold:

$$\begin{aligned} I_{inj}(0) &= I_{stim} - I_{post}(0) \\ &= I_{th} \end{aligned} \quad \text{(SI.5)}$$

Inserting Eq.SI.3 and SI.2 into Eq.SI.1 gives the frequency of phasic synchronization as:

$$\frac{1}{f_p} = x\left(1-\frac{\tau_u}{\tau_d}\right) - \tau_u \ln\left(1-\frac{I_0}{I_{stim}-I_{th}}\left[1-\exp\left(-\frac{x}{\tau_d}\right)\right]\right) \quad \text{(SI.6)}$$

Since $\tau_d \approx \tau_u$:

$$\boxed{\frac{1}{f_p} = -\tau_u \ln\left(1-\frac{I_0}{I_{stim}-I_{th}}\left[1-\exp\left(-\frac{W-d}{\tau_d}\right)\right]\right)} \quad \text{(SI.7)}$$

Frequency of phase-locked oscillations (red curve in Fig.1(d))

<u>Supplementary Discussion II</u>: Counting the total number of attractors in a network of N neurons

One now counts the number of periodic trajectories that may be realized in a network of *N* neurons. The *N* neurons discharge within a period that, in general, contains *M* inter-spike intervals (ISI) of duration *T*/*M*. Hence *M* neurons discharge during each ISI whilst *N*-*M* neurons discharge in phase with another neuron. An example for *N*=7, *M*=4 is given in Figure S11.

A sequence of spikes is described by a list of positive integers $\{k_i\}$ which states *the number of neurons that discharge in ISI* i where $i = 1,2,...M$. There are k_1 neurons discharging in ISI #1, k_2 neurons in ISI #2, … k_M neurons in ISI #*M.* This list satisfies the sum rule:

$$\sum_{i=1}^{M} k_i = N \tag{SI.8}$$

One also defines the dual of this list $\{n_j\}$ which states the *number of ISIs in each period which contain* j *spikes* where $j = 1,2,..M$. There are n_1 ISIs which have a single spike, n_2 ISIs with 2 spikes, … n_N ISIs with *N* spikes. The dual list satisfies the sum rule:

$$\sum_{j=1}^{N} n_j = M \tag{SI.9}$$

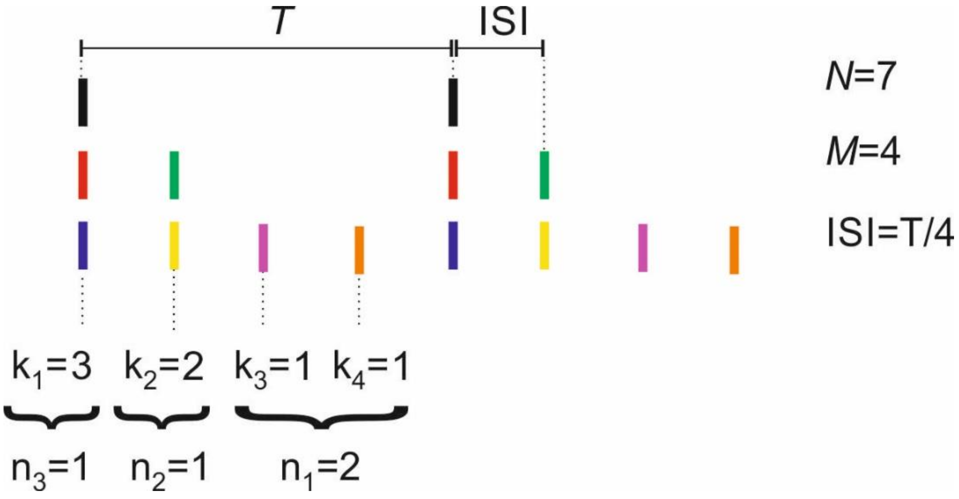

Figure S11: Example of partially synchronized oscillation pattern

The 7 neurons discharge in a sequence with *M*=4 ISIs per period and an ISI duration of $T/4$. The spike arrangement is $(k_1, k_2, k_3, k_4) = (3, 2,1,1)$. The dual list is $(n_1, n_2, n_3, n_4, n_5, n_6, n_7) = (2,1,1,0,0,0,0)$. The number of waveforms that may be obtained with spike groupings $(n_1, n_2, n_3, n_4, n_5, n_6, n_7)$ is $M!/(n_1!n_2!n_3!n_4!n_5!n_6!n_7!) = 12$. The number of waveforms is $N!/(k_1!k_2!k_3!k_4!) = 420$.

1. Number of waveforms of undiscernible spikes

Spike groupings can be rearranged in a number of ways over the *M* inter-spike intervals which all realize the frequency spectrum $(n_1, n_2, ... n_N)$. Each rearrangement gives a "waveform" which spike groupings appear in a particular sequence. For now spikes are considered undiscernible. The number of possible waveforms that may be constructed from the frequency spectrum of spike groupings $n_1, n_2, ..., n_N$ is:

$$W_N^M\{n_1,n_2...,n_N\} = \binom{M}{n_1}\times\binom{M-n_1}{n_2}\times\binom{M-n_1-n_2}{n_3}\times...\times\binom{M-n_1...-n_{N-1}}{n_N}$$
$$= \frac{M!}{M!(M-n_1)!}\times\frac{(M-n_1)!}{n_2!(M-n_1-n_2)!}\times...\frac{(M-n_1...-n_{N-1})!}{n_N!(M-n_1-n_2...-n_N)!}$$
$$= \frac{M!}{n_1!n_2!...n_N!(M-M)!} \qquad \text{(SI.10)}$$
$$= \frac{M!}{n_1!n_2!...n_N!}$$

The number of waveforms of undiscernible spikes is the multinomial coefficient:

$$W_N^M\{n_1,n_2,...n_N\} = \binom{M}{n_1,\ n_2,\ ...,n_N} \qquad \text{(SI.11)}$$

2. Number of spike arrangements within each waveform

For each list $\{k_1,k_2,...k_M\}$, there are $A_N^M\{k_1,k_2,...k_M\}$ possible ways of distributing *N* discernible spikes over the *M* ISIs. This is the number of ways of distributing k_1 spikes out of *N* in ISI #1, k_2 spikes out of *N*-k_1 in ISI #2 ....and k_M spikes in ISI #*M*. $A_N^M\{k_1,k_2,...k_M\}$ is given by:

$$A_N^M\{k_1,k_2...k_M\} = \binom{N}{k_1}\times\binom{N-k_1}{k_2}\times\binom{N-k_1-k_2}{k_3}\times...\times\binom{N-k_1...k_{M-1}}{k_M}$$
$$= \frac{N!}{k_1!(N-k_1)!}\times\frac{(N-k_1)!}{k_2!(N-k_1-k_2)!}\times...\frac{(N-k_1...-k_{M-1})!}{k_M!(N-k_1-k_2...-k_M)!}$$
$$= \frac{N!}{k_1!k_2!k_3!...k_M!(N-N)!}$$
$$= \frac{N!}{k_1!k_2!k_3!...k_M!}$$

The number of spike arrangements per waveform is the multinomial coefficient:

$$A_N^M\{k_1,...,k_M\} = \binom{N}{k_1,\ k_2,\ ...,k_M} \qquad \text{(SI.12)}$$

3. Total number of attractors

This is obtained by multiplying the number of waveforms (Eq.SI.11) with the number of trajectories per waveform (Eq.SI.12) and summing over the partition of integer N. This partition corresponds to the decreasing list $k_1 \geq k_2 \geq \ldots \geq k_M$ which satisfies to the condition $k_1 + k_2 + \ldots + k_M = N$. For example, the partition of $N = 5$ is:

k_1	k_2	k_3	k_4	k_5
**1**	**1**	**1**	**1**	**1**
**2**	**1**	**1**	**1**	
**2**	**2**	**1**		
**3**	**1**	**1**		
**3**	**2**			
**4**	**1**			
**5**				

The total *number of possible trajectories* in an *N*-neuron network is:

$$\sum_{k_1+k_2+\ldots+k_M=N} \binom{M}{n_1\, n_2 \ldots n_N} \binom{N}{k_1\, k_2 \ldots k_M}$$

This however includes trajectories which are equivalent by *M*-cyclical permutation of indices. The *number of cyclically independent trajectories* is thus obtained by dividing this number by *M*. The number of cyclically independent trajectories hence dynamic attractors in an *N*-neuron network is:

$$T_N = \sum_{k_1+k_2+\ldots+k_M=N} \underbrace{\binom{M-1}{n_1, n_2, \ldots, n_N}}_{w_N^M = W_N^M / M} \underbrace{\binom{N}{k_1, k_2, \ldots, k_M}}_{A_N^M} \qquad \text{(SI.13)}$$

4. Calculation of the number of attractors for inhibitory networks with 3-6 neurons

N=3

M	ISI	k_1	k_2	k_3	n_1	n_2	n_3	$w_N^M\{\vec{n}\}$	$A_N^M\{\vec{k}\}$	$w_N^M \times A_N^M$
3	*T*/3	**1**	**1**	**1**	**3**	0	0	**1/3**	**6**	**2**
2	*T*/2	**2**	**1**	0	**1**	**1**	0	**1**	**3**	**3**
1	*T*/1	**3**	0	0	0	0	**1**	**1**	**1**	**1**
									**Number of cyclical trajectories/point attractors:**	**6**

N=4

M	ISI	k_1	k_2	k_3	k_4	n_1	n_2	n_3	n_4	$w_N^M\{\vec{n}\}$	$A_N^M\{\vec{k}\}$	$w_N^M \times A_N^M$
4	*T*/4	1	1	1	1	4	0	0	0	1/4	24	6
3	*T*/3	2	1	1	0	2	1	0	0	1	12	12
2	*T*/2	3	1	0	0	1	0	1	0	1	4	4
2	*T*/2	2	2	0	0	0	2	0	0	1/2	6	3
1	*T*/1	4	0	0	0	0	0	0	1	1	1	1
											**Number of cyclical trajectories/point attractors:**	**26**

N=5

M	ISI	k_1	k_2	k_3	k_4	k_5	n_1	n_2	n_3	n_4	n_5	$w_N^M\{\vec{n}\}$	$A_N^M\{\vec{k}\}$	$w_N^M \times A_N^M$
5	*T*/5	1	1	1	1	1	5	0	0	0	0	1/5	120	24
4	*T*/4	2	1	1	1	0	3	1	0	0	0	1	60	60
3	*T*/3	3	1	1	0	0	2	0	1	0	0	1	20	20
3	*T*/3	2	2	1	0	0	0	2	1	0	0	1	30	30
2	*T*/2	4	1	0	0	0	1	0	0	1	0	1	5	5
2	*T*/2	3	2	0	0	0	0	1	1	0	0	1	10	10
1	*T*/1	5	0	0	0	0	0	0	0	0	1	1	1	1
													**Number of cyclical trajectories/point attractors:**	**150**

N=6

M	ISI	k_1	k_2	k_3	k_4	k_5	k_6	n_1	n_2	n_3	n_4	n_5	n_6	$w_N^M\{\vec{n}\}$	$A_N^M\{\vec{k}\}$	$w_N^M \times A_N^M$
6	*T*/6	1	1	1	1	1	1	6	0	0	0	0	0	1/6	720	120
5	*T*/5	2	1	1	1	1	0	4	1	0	0	0	0	1	360	360
4	*T*/4	3	1	1	1	0	0	3	0	1	0	0	0	1	120	120
4	*T*/4	2	2	1	1	0	0	2	2	0	0	0	0	3/2	180	270
3	*T*/3	4	1	1	0	0	0	2	0	0	1	0	0	1	30	30
3	*T*/3	3	2	1	0	0	0	1	1	1	0	0	0	2	60	120
3	*T*/3	2	2	2	0	0	0	0	0	3	0	0	0	1/3	90	30
2	*T*/2	5	1	0	0	0	0	1	0	0	0	1	0	1	6	6
2	*T*/2	4	2	0	0	0	0	0	1	0	1	0	0	1	15	15
2	*T*/2	3	3	0	0	0	0	0	0	2	0	0	0	1/2	20	10
1	*T*/1	6	0	0	0	0	0	0	0	0	0	0	1	1	1	1
															**Number of cyclical trajectories/point attractors:**	**1082**

Capacity of gap junction networks:
Neurons are only able to discharge sequentially which implies *M*=*N* and $k_1 = k_2 = \ldots = k_N = 1$. In this particular case, Eq.SI.13 gives:

$$T_N = \sum_{k_1=k_2=\ldots=k_N=1} \binom{N-1}{n_1, n_2, \ldots, n_N} \binom{N}{k_1, k_2, \ldots, k_M} = \frac{N!}{N} = (N-1)!$$

which is the well-known capacity of a winnerless network[4].

[4] M. Rabinovich, A. Volkovskii, P. Lecanda, R. Huerta, H.D.I. Abarbanel, G. Laurent, *Phys. Rev. Lett.* **87**, 068102 (2001)
